# In-resin CLEM of Epon-embedded cells using proximity labeling

**DOI:** 10.1038/s41598-022-15438-6

**Published:** 2022-07-01

**Authors:** Takahito Sanada, Junji Yamaguchi, Yoko Furuta, Soichiro Kakuta, Isei Tanida, Yasuo Uchiyama

**Affiliations:** 1 Department of Cellular and Molecular Neuropathology, Juntendo University Graduate School of Medicine, Tokyo, Japan; 2 Laboratory of Morphology and Image Analysis, Biomedical Research Core Facilities, Juntendo University Graduate School of Medicine, Tokyo, Japan

**Keywords:** Mitochondria, Cellular imaging, Nucleus

## Abstract

Biotin ligases have been developed as proximity biotinylation enzymes for analyses of the interactome. However, there has been no report on the application of proximity labeling for in-resin correlative light-electron microscopy of Epon-embedded cells. In this study, we established a proximity-labeled in-resin CLEM of Epon-embedded cells using miniTurbo, a biotin ligase. Biotinylation by miniTurbo was observed in cells within 10 min following the addition of biotin to the medium. Using fluorophore-conjugated streptavidin, intracellular biotinylated proteins were labeled after fixation of cells with a mixture of paraformaldehyde and glutaraldehyde. Fluorescence of these proteins was resistant to osmium tetroxide staining and was detected in 100-nm ultrathin sections of Epon-embedded cells. Ultrastructures of organelles were preserved well in the same sections. Fluorescence in sections was about 14-fold brighter than that in the sections of Epon-embedded cells expressing mCherry2 and was detectable for 14 days. When mitochondria-localized miniTurbo was expressed in the cells, mitochondria-like fluorescent signals were detected in the sections, and ultrastructures of mitochondria were observed as fluorescence-positive structures in the same sections by scanning electron microscopy. Proximity labeling using miniTurbo led to more stable and brighter fluorescent signals in the ultrathin sections of Epon-embedded cells, resulting in better performance of in-resin CLEM.

## Introduction

Correlative light and electron microscopy (CLEM) is a method for the correlation of fluorescence microscopy with electron microscopy^1^. In traditional CLEM, fluorescent signals in the cells are first obtained after fixation of the cells with paraformaldehyde (and glutaraldehyde)^2^. Thereafter, the cells are fixed with a mixture of paraformaldehyde and glutaraldehyde, stained with osmium tetroxide, dehydrated with ethanol, and embedded into resins (mainly epoxy resins). After preparation of ultrathin sections (50–100 nm thickness) of resin-embedded samples, electron microscopic images are obtained. Therefore, there are unavoidable chemical and physical distortions between the fluorescent and electron microscopic images.

To overcome these limitations of CLEM, in-resin CLEM was developed^3–5^. In-resin CLEM aims to obtain fluorescent and electron microscopic images from the same thin sections of resin-embedded samples. However, there are some limitations of in-resin CLEM of ‘Epon’-embedded samples with osmium tetroxide staining^3,6^. Epon-embedding is a robust electron microscopic technique to preserve intracellular ultrastructures in the cells for electron microscopy, and osmium tetroxide staining is important to visualize membranous structures using electron microscopy. However, epoxy resins have autofluorescence, and osmium tetroxide diminishes the fluorescent intensity of many fluorescent proteins and fluorophores^7,8^.

Recently, some fluorescent proteins (mEosEM, mWasabi, CoGFP variant 0, mCherry2, and mKate2-GGGGSGL) showed resistance against osmium tetroxide staining^6,7,9,10^, and two color in-resin CLEM of Epon-embedded cells using these green and red fluorescent proteins was achieved^7,10^. However, there are weaknesses in the retention of fluorescent intensity of fluorescent proteins in the Epon-embedded samples^10^. Fluorescent intensities in the Epon-embedded samples tend to be weakened and decrease significantly within a week at room temperature. Therefore, a brighter and more stable labeling method for in-resin CLEM of Epon-embedded cells is required.

To settle this issue, we focused on the proximity labeling enzymes and the biotin-streptavidin reaction^11–16^. The proximity labeling methods using engineered ascorbate peroxidases and biotin ligases are mainly developed to analyze protein–protein interactions in living cells^12^. APEX2, one of the engineered ascorbate peroxidases, generates biotin phenoxy radicals in the presence of hydrogen peroxide and biotin phenol during proximity labeling. Based on this reaction and biotin–avidin interaction, APEX2 in the cells was detected by a fluorescence microscope with fluorophore-conjugated streptavidin and detected by electron microscopy using an enzymatic reaction with 3,3ʹ-diaminobenzidine converting into an insoluble osmiophilic polymer^12,13^. Until recently, however, because no study has reported the application of engineered ascorbate peroxidases for in-resin CLEM of Epon-embedded cells.

Biotin ligase mediates the conjugation of proximity proteins and itself with biotin in living cells^13–16^. Some engineered biotin ligases including BASU, TurboID, miniTurbo, and AirID are able to label proximal proteins with biotin within 10 min without cell toxicity, whereas the other biotin ligases (BirA, its mutant, BioID, and BioID2) require more than 10 h for proximity labeling^15–18^. In general, excess intracellular accumulation of biotinylated proteins results in cytotoxicity^15^. miniTurbo has a lower activity than TurboID or AirID, which leads to mild intracellular biotinylation as compared with the others, resulting in minimizing cytotoxicity. Biotinylated proteins modified by a biotin ligase in the cells were detected by fluorescence microscopy using a fluorophore-conjugated streptavidin, but none was analyzed by electron microscopy or in-resin CLEM of Epon-embedded samples. In this study, we focused on miniTurbo as a biotin ligase for proximity labeling in in-resin CLEM, and applied it for in-resin CLEM of Epon-embedded cells.

## Results

### Biotinylated proteins by a biotin ligase in cells were detected after osmium tetroxide staining

We first investigated the conditions for biotinylation of intracellular proteins by miniTurbo. The miniTurbo protein was expressed well, and biotinylated proteins increased in a time-dependent manner, which coincides with the previous report (Supplementary Fig. 1A and B)^15^. We generated a miniTurbo fused with a targeting signal sequence for the mitochondrial outer membrane^7,19–21^. mtActA-mTurbo. Similar biotinylation of proteins was detected when mtActA-mTurbo was expressed in the cells (Supplementary Fig. 1A and B). Intracellular biotinylated proteins in cells expressing mtActA-mTurbo were significantly colocalized with Tom20, a mitochondrial marker (the Pearson correlation coefficient value ± SEM was 0.82 ± 0.012, n = 12) (Supplementary Fig. 2).

**Figure 1:**
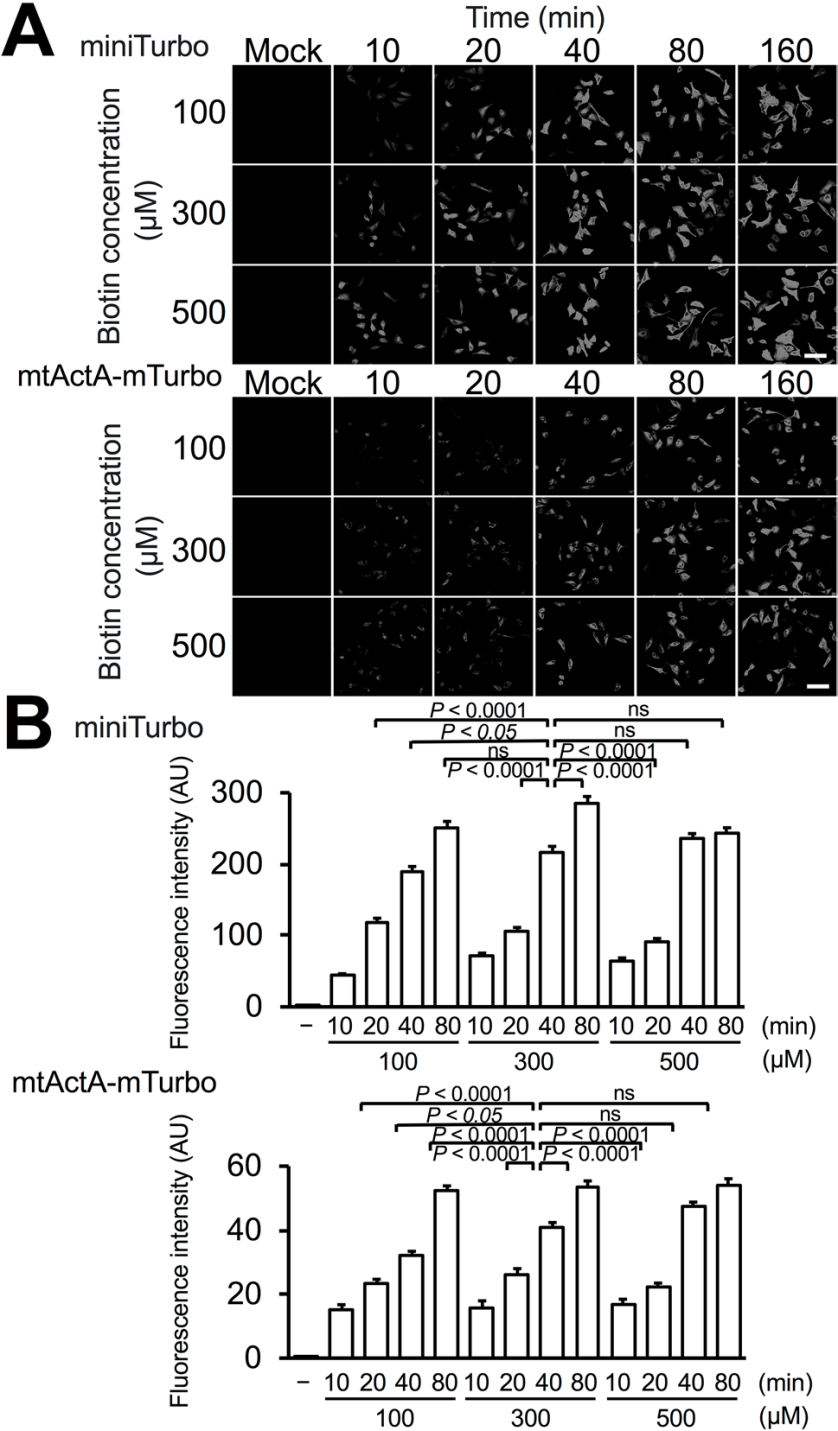
Fluorophore-conjugated streptavidin reacted with biotinylated proteins in cells after fixation with glutaraldehyde-containing mixture. (**A**) HeLa cells expressing miniTurbo (**A**, upper panel) and mtActA-mTurbo fusion protein (**A**, lower panel) were incubated in the presence of biotin (100, 300, and 500 μM) for the indicated times (10–160 min). After permeabilization, DyLight 549-streptavidin was reacted with the biotinylated proteins. Fluorescent images were obtained using a BZ-X810 fluorescence microscope (CCD monochrome camera, Nikon CFI Plan Apochromat × 40 lens, gain + 16 dB) using a filter set (excitation: 520–570 nm, dichroic mirror: 565 nm long pass, emission: 535–675 nm). (**B**) Fluorescent intensity in (**A**) was evaluated by ImageJ software. Error bars indicate the standard errors. Scale bar, 200 µm. *P value < 0.05, ****P value < 0.0001, *ns* not significant.

In general, when intracellular biotinylated proteins in cells are analyzed by fluorescence microscopy, the cells are fixed with formaldehyde or paraformaldehyde. However, for electron microscopy, it is important that cells are fixed with a mixture of paraformaldehyde and glutaraldehyde (or glutaraldehyde alone) to preserve intracellular structures. In the presence of glutaraldehyde (0.1–2.5%) in a fixation mixture, some affinity-reactions including antigen–antibody reactions are significantly inhibited. We investigated the issue of whether fluorophore-conjugated streptavidin was able to react with biotinylated proteins by miniTurbo and mtActA-mTurbo after fixation with a mixture of paraformaldehyde and glutaraldehyde. When in-resin CLEM using red fluorescent proteins was performed, red autofluorescence of 100-nm thin sections of Epon-embedded samples was lower than for blue and green autofluorescence^7,9,10^. Therefore, we employed a red fluorophore, DyLight 549, for in-resin CLEM. After biotinylation, the HeLa cells expressing miniTurbo and mtActA-mTurbo were fixed with a glutaraldehyde-containing fixative mixture (4% paraformaldehyde and 0.25% glutaraldehyde). After permeabilization, DyLight 549-conjugated streptavidin reacted with biotinylated proteins. Observation under a fluorescence microscope (Fig. 1), indicated that biotinylated proteins reacted with the fluorophore-conjugated streptavidin after fixation with the mixture of paraformaldehyde and glutaraldehyde. The fluorescent intensities of biotinylated proteins by miniTurbo and mtActA-mTurbo at 10–20 min after the addition of biotin (100, 300, 500 µM) were less than half of intensities at 80 min. We used the treatment of 300 µM biotin for 40 min in the following CLEM experiments.

**Figure 2:**
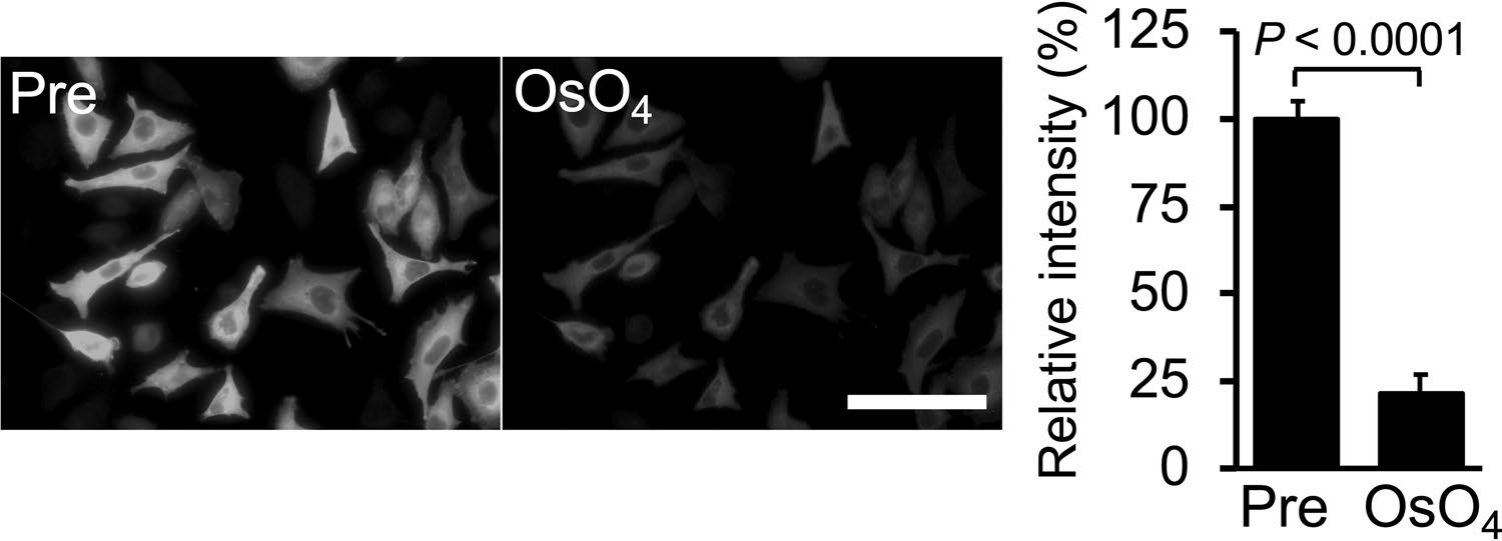
Fluorescent signals of biotinylated proteins were resistant to osmium tetroxide and Epon-embedding. (**A**) Changes of fluorescent signals following treatment with 2% osmium tetroxide at 4 °C for 30 min. Cells containing fluorophore-labeled biotinylated proteins were incubated in 2% osmium tetroxide at 4 °C for 30 min. Fluorescent images were obtained before (**Pre**) and after (**OsO**_**4**_) treatment with osmium tetroxide. The fluorescent intensity in (**A**) was evaluated by ImageJ software. Relative intensity is shown when the fluorescent intensity before the treatment as 100%. Scale bar, 100 μm. **P value < 0.01, ****P value < 0.0001. Fluorescent images were obtained under the same conditions for mTurbo using a BZ-X810 fluorescence microscope (CCD monochrome camera, Nikon CFI Plan Apochromat × 40 lens, gain + 16 dB, on-chip binning off, exposure time 1/2 s) using a filter set (excitation: 520–570 nm, dichroic mirror: 565 nm long pass, emission: 535–675 nm).

Treatment with osmium tetroxide diminishes the fluorescent intensity of many fluorescent proteins and fluorophores, although osmium tetroxide staining is essential to visualize intracellular membranous structures by electron microscopy. We investigated the issue of whether osmium tetroxide staining decreased the fluorescent intensity of fluorophore-labeled biotinylated proteins. After fixation with the glutaraldehyde-containing mixture, cells expressing miniTurbo were stained with the fluorophore-conjugated streptavidin and were incubated in 2% osmium tetroxide at 4 °C for 30 min. After osmium tetroxide staining, fluorescent signals were detected with a fluorescence microscope under the same acquisition settings (Fig. 2). The fluorescent intensity of fluorophore in the cells was about 78% drop by osmium tetroxide staining but strong enough to be detected by a BZ-X810 fluorescence microscope.

### In-resin CLEM of Epon-embedded cells expressing miniTurbo was performed

**Figure 3:**
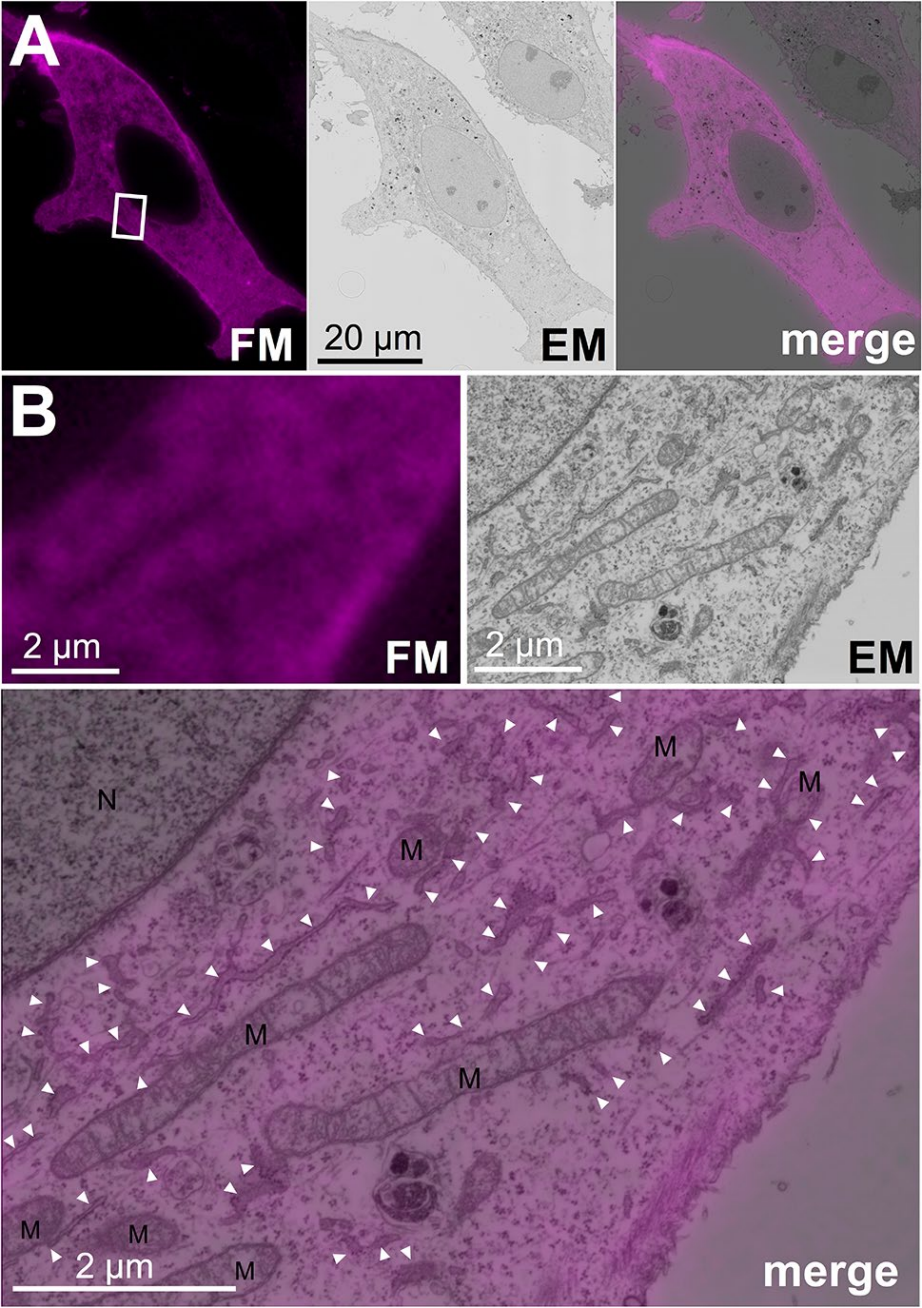
In-resin CLEM of Epon-embedded cells was performed using miniTurbo. Thin sections (100 nm) of Epon-embedded cells expressing miniTurbo (magenta pseudo color) were prepared. Fluorescent images (**FM**) were obtained in the presence of TUK Solution for multicolor using a BZ-X810 fluorescence microscope (CCD monochrome camera, Nikon CFI Plan Apochromat × 100 Oil lens, gain + 16 dB) using a filter set (excitation: 520–570 nm, dichroic mirror: 565 nm long pass, emission: 535–675 nm) for red fluorescent probes. Electron microscopic images (**EM**) were obtained with a Helios NanoLab 660 scanning electron microscope (a backscattered electron detector at a voltage of 2.0 kV and a current of 0.4 nA). The “**merge**” is the merged image of the fluorescence (**FM**) and electron microscopic images (**EM**). The images in (**B**) indicate 10x (**FM** and **EM**) and × 20 (**merge**) magnification of the respective images corresponding to the boxed area in the **merge** image in (**A**). M, mitochondria; N, nucleus; arrowheads, endoplasmic reticulum.

For electron microscopy, cells are further dehydrated ethanol and embedded in epoxy resins^2,10^. These treatments tend to diminish the fluorescent intensity of many fluorophores. If fluorophore-labeled biotinylated proteins in the cells are resistant to dehydration with ethanol and polymerization of epoxy resins, proximity labeled in-resin CLEM of Epon-embedded cells will be achieved. To investigate the fluorescence of fluorophore-labeled biotinylated proteins in the 100-nm thin sections of Epon-embedded cells, cells were dehydrated with a graded series of ethanol (50%, 70%, 90%, 95%, and 100%), stained with osmium tetroxide, and embedded in epoxy resin at 60 °C for 72 h. After the preparation of 100-nm thin sections of the Epon-embedded cells, the fluorescence of the sections was investigated. Fluorescence of biotinylated proteins was well detected in the cells of thin sections (Fig. 3, FM). Ultrastructures of cells in the same section were also analyzed by a Helios NanoLab 660 scanning electron microscope using a circular backscatter detector at a voltage of 2.0 kV and a current of 0.4 nA (Fig. 3, EM). The electron microscopic image was well correlated with the fluorescent image (Fig. 3, merge). Intracellular ultrastructures including mitochondria and endoplasmic reticulum in the cells of the section were well preserved under these conditions. These results indicated that in-resin CLEM of Epon-embedded cells using miniTurbo was achieved.

### Fluorescent signals in ultrathin sections of in-resin CLEM using a biotin ligase were brighter and more stable than those with a fluorescent protein, mCherry2

**Figure 4:**
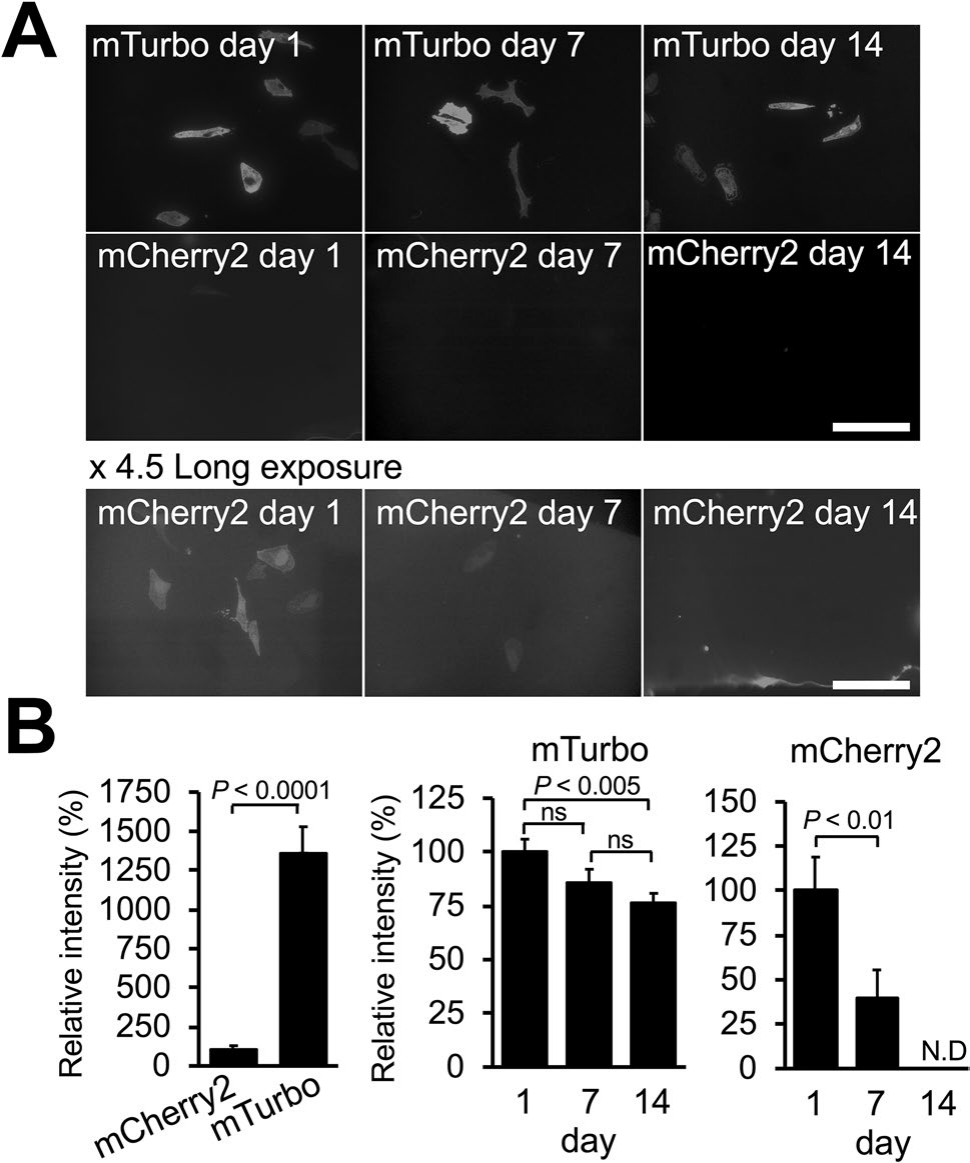
Fluorescent signals in ultrathin sections of in-resin CLEM using a biotin ligase were brighter and more stable than those with a fluorescent protein, mCherry2. (**A**) Time-dependent decreases in fluorescent signals of fluorophore-labeled biotinylated proteins in the 100-nm thin sections of Epon-embedded cells. Fluorescent images of biotinylated proteins in the sections of Epon-embedded cells were obtained by fluorescence microscopy (**upper** panels, **mTurbo**). Fluorescent images of mCherry2 in the sections of Epon-embedded cells expressing **mCherry2** are shown in the **middle** and **lower** panels. In the **middle** panels, fluorescent images were obtained under the same conditions for mTurbo using a BZ-X810 fluorescence microscope (CCD monochrome camera, Nikon CFI Plan Apochromat × 40 lens, gain + 16 dB, 2 × 2 on-chip binning, exposure time 1/1.5 s) using a filter set (excitation: 520–570 nm, dichroic mirror: 565 nm long pass, emission: 535–675 nm). Images in the **lower** panels (**Long exposure**) were 4.5 × longer exposure of those in the **middle** panels. Note that fluorescence of proximity-labeled Epon-embedded cells (**mTurbo**) was brighter than that of **mCherry2**-expressed Epon-embedded cells. (**B**) Evaluation of brightness and stability in the 100-nm thin sections of the proximity-labeled Epon-embedded cells. The relative intensity of fluorescence mediated by mTurbo was compared with that of mCherry2 (**left** graph). Time-dependent decreases of fluorescence in the thin sections of **mTurbo**- and **mCherry2**-expressed Epon-embedded cells were evaluated at 1, 7, and 14 days after preparation of the sections (**middle** and **right** graphs). Relative intensity of fluorescence is shown such that the intensity at day 1 is estimated as 100%. Error bars indicate standard errors.

Fluorescent signals of in-resin CLEM with miniTurbo are derived from the brightness of the fluorophore, multiple biotinylation of proteins proximity labeled by miniTurbo, and tetramer formation of streptavidin, whereas fluorescent signals of in-resin CLEM using fluorescent protein are derived from the active fluorescent protein only. Given these factors, it is possible that fluorescent signals in this in-resin CLEM using miniTurbo will be brighter than those of in-resin CLEM using fluorescent protein. To clarify this possibility, we compared the fluorescent intensity of in-resin CLEM using miniTurbo with that of in-resin CLEM using mCherry2, because mCherry2 is one of the brightest fluorescent proteins suitable for in-resin CLEM of in Epon-embedded samples^7,10^. HeLa cells expressing mCherry2 and miniTurbo were postfixed with osmium and embedded in Epon 812 resins. Fluorescent signals in each section of Epon-embedded cells were investigated with a fluorescence microscope under the same acquisition settings (Fig. 4A and B, day 1). The fluorescent intensity in the 100-nm thin sections of Epon-embedded cells expressing miniTurbo was about 14-fold higher than that in the sections of cells expressing mCherry2.

The stability of fluorescent signals is an important factor for in-resin CLEM. Fluorescent signals derived from fluorescent proteins in Epon-embedded cells decrease significantly within a week^10^. If a fluorophore in Epon-embedded cells is more stable than fluorescent proteins in Epon-embedded cells, in-resin CLEM of Epon-embedded cells using miniTurbo has a great advantage in fluorescence microscopic analyses compared with that using mCherry2. To investigate the stability of fluorescent signals of proximity-labeled in-resin CLEM of Epon-embedded cells, we examined the time-dependent changes of fluorescent signals in 100-nm thin sections of Epon-embedded cells expressing miniTurbo (Fig. 4A and B, days 7 & 14). The fluorescent signals in sections of cells expressing miniTurbo were stable at 7 days after this preparation and were about 23% drop on day 14. In contrast, the fluorescent signals of mCherry2 were about 60% drop at 7 days after the preparation, and no signals were detected on day 14. These results indicated that fluorescent signals in this in-resin CLEM using proteins biotinylated by miniTurbo and fluorophore-conjugated streptavidin were more stable than those using mCherry2.

### In-resin CLEM of mitochondria and nucleus in Epon-embedded cells using proximity labeling was performed

**Figure 5:**
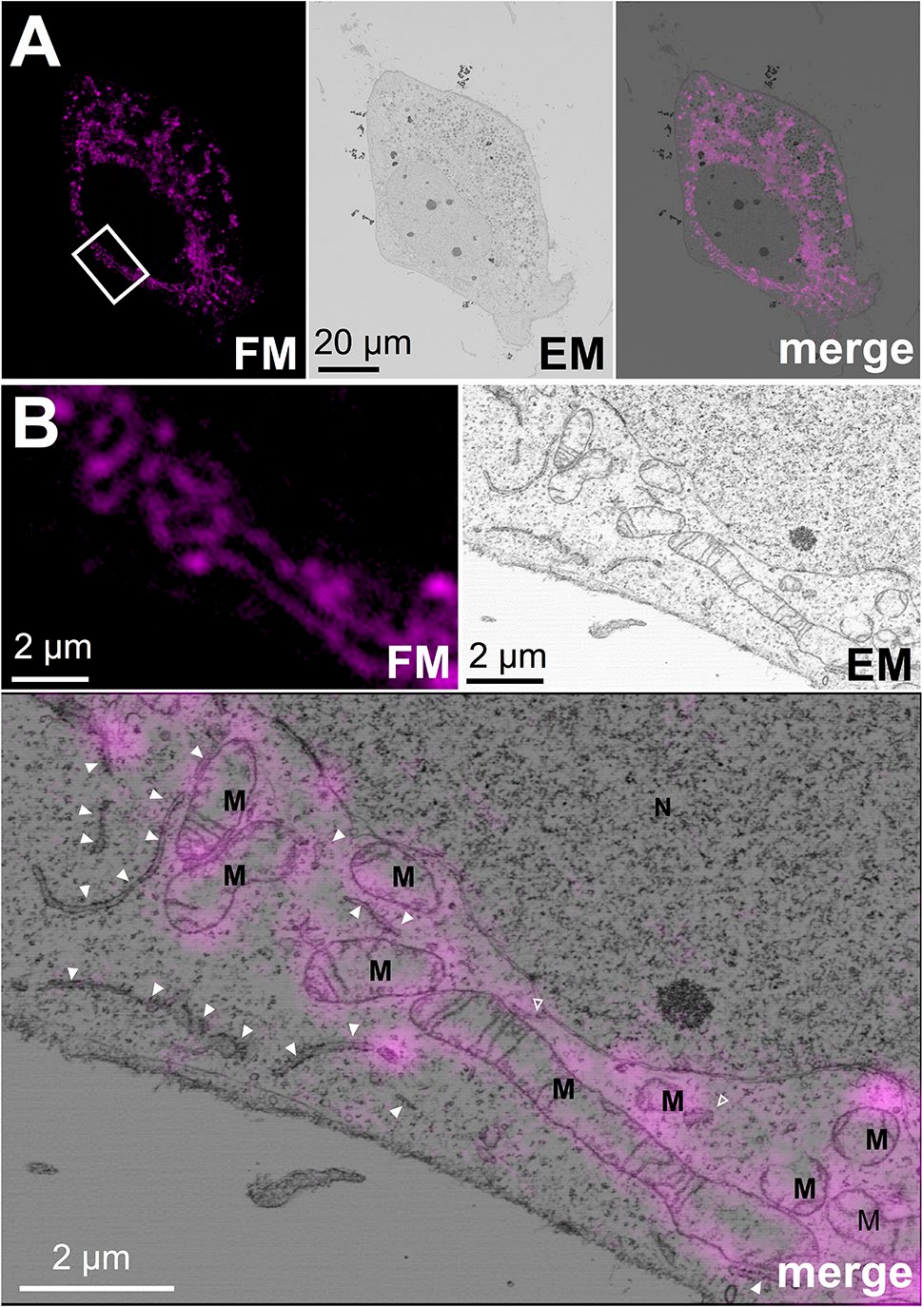
In-resin CLEM of mitochondria of Epon-embedded cells was performed using mtActA-mTurbo. Thin sections (100 nm) of Epon-embedded cells expressing mtActA-mTurbo (magenta pseudo color) were prepared. Fluorescent images (**FM**) and electron microscopic images (**EM**) were obtained as described in Fig. 3. The “**merge**” is a merged image of the fluorescence (**FM**) and electron microscopic images (**EM**). The images in (**B**) indicate × 12.5 (**FM** and **EM**) and × 25 (**merge**) magnification of the respective images corresponding to the boxed area in the **merge** image in (**A**). *M* mitochondria, *N* nucleus; arrowheads, endoplasmic reticulum.

We investigated the application of proximity labeling with miniTurbo for in-resin CLEM of intracellular organelles in the Epon-embedded cells. Proteins in HeLa cells expressing mtActA-mTurbo were biotinylated by addition of biotin to the medium and were fixed. Biotinylated proteins in the cells were reacted with DyLight 549-conjugated streptavidin, and cells were embedded in the Epon resins as described above. After preparation of 100-nm thin sections of Epon-embedded cells, fluorescent signals were observed by confocal fluorescence microscopy. The intracellular fluorescent signals in the sections showed a mitochondrial-like distribution (Fig. 5, FM). Electron microscopy of the same sections revealed that the ultrastructures of mitochondria were observed and well preserved in the fluorescent signal-positive areas (Fig. 5, EM). Fluorescent signals derived from biotinylated proteins were well correlated with mitochondrial structures in the electron microscopic images (Fig. 5, merge). To quantify the correlation between fluorescent signal-positive areas in the fluorescent microscopic images and ultrastructures of mitochondrial outer membrane in the electron microscopic images, we performed a point counting analysis^22–24^. Fluorescent signals were mainly correlated with the ultrastructures of mitochondrial outer membranes (about 95%), while a few were correlated with the endoplasmic reticulum (9.5%) and nucleus (2.7%) (Supplementary Fig. 3). These results indicated that in-resin CLEM of mitochondria in Epon-embedded cells expressing mtActA-mTurbo was achieved.

**Figure 6:**
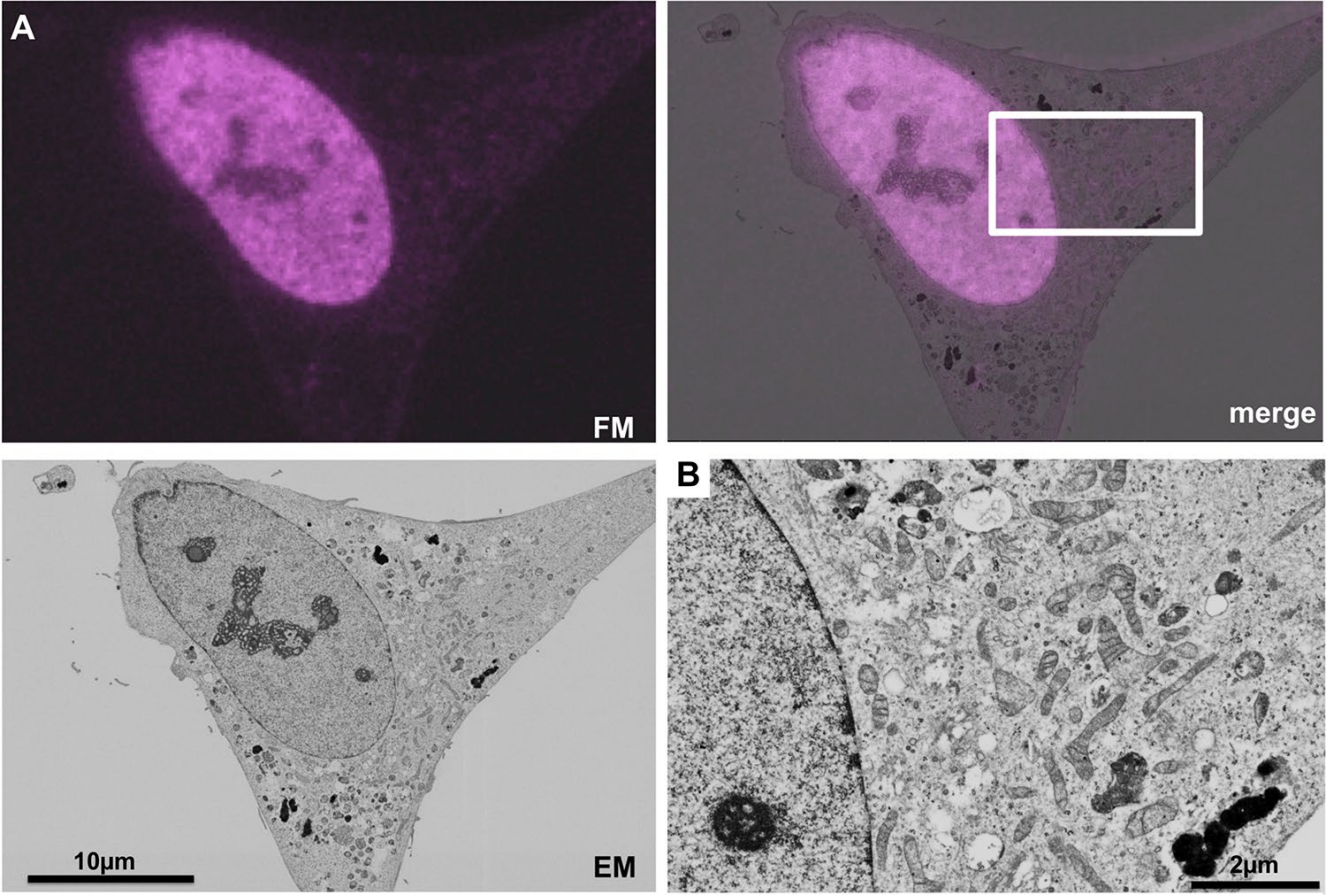
In-resin CLEM of nuclei of Epon-embedded cells was performed using **H2B-mTurbo.** Thin sections of Epon-embedded cells expressing H2B-mTurbo (magenta pseudo color) were prepared. Fluorescent images (**FM**) and electron microscopic images (**EM**) were obtained as described in Fig. 3. The “**merge**” is a merged image of the fluorescence (**FM**) and electron microscopic images (**EM**). The image in (**B**) indicates × 4 magnification of the electron microscopic image corresponding to the boxed area in the **merge** image in (**A**).

Next, we constructed an expression vector for H2B-mTurbo, which was the fusion protein of histone H2B and miniTurbo. HeLa cells expressing H2B-mTurbo were incubated with biotin, fixed, and stained with DyLight 549-conjugated streptavidin. After preparation of Epon-embedded cells, 100-nm thin sections were prepared. A fluorescent image was detected by fluorescence microscopy. Fluorescence in the section showed a nuclear-like localization (Fig. 6A). An electron microscopic image in the same section was obtained by scanning electron microscopy. The correlation of fluorescent and electron microscope images revealed that the fluorescent signal was observed in the nucleus (Fig. 6A), and that intracellular ultrastructures including nucleus and mitochondria were well preserved (Fig. 6B). These results indicated that in-resin CLEM of nuclei in Epon-embedded cells expressing H2B-mTurbo was also achieved.

## Discussion

We have, for the first time, applied proximity labeling of biotinylated proteins with a biotin ligase for in-resin CLEM of Epon-embedded cells. Biotinylation of proximity proteins by miniTurbo and its fusion proteins occurs within 10 min in vivo following the addition of biotin to the culture medium. Intracellular biotinylated proteins were able to react with fluorophore-conjugated streptavidin after fixation of the cells in the presence of glutaraldehyde. The fluorophore used in this study was resistant to osmium tetroxide staining, dehydration with ethanol, and polymerization of Epon resins. Fluorescent signals were detected in the 100-nm thin sections of Epon-embedded cells. Electron microscopic images were obtained of the same section, indicating that in-resin CLEM of Epon-embedded cells expressing miniTurbo and its fusion proteins was performed. Fluorescent signals of proximity-labeled in-resin CLEM using miniTurbo were brighter and more stable than those of in-resin CLEM using a fluorescent protein, mCherry2.

Application of proximity labeling with miniTurbo for in-resin CLEM of Epon-embedded cells has great advantages compared with that of fluorescent proteins for in-resin CLEM^10^. Fluorescent signals of DyLight 549 in the 100-nm sections of Epon-embedded cells expressing miniTurbo were about 14-fold brighter than those using mCherry2. Fluorescent signals in the thin sections of proximity-labeled Epon-embedded cells decreased only to about 64% at 14 days after preparation of ultrathin sections, whereas fluorescent signals in the section of Epon-embedded cells expressing mCherry2 decreased to non-detectable levels at 14 days. Given that thinner sections led to weaker fluorescent signals, miniTurbo-based in-resin CLEM will be better for analyzing the intracellular localization of minor proteins or small structures of organelles.

The fluorescent intensity and stability of this in-resin CLEM using miniTurbo were dependent on the amounts of biotinylated proteins, tetramer formation of streptavidin, and properties of the fluorophore. In this study, we employed red fluorophores, because red autofluorescence in the thin sections of Epon-embedded cells was low enough to be ignored compared with red fluorescence of these fluorophores. We performed in-resin CLEM using miniTurbo with green fluorophore (DyLight 488, Alexa Fluor 488, fluorescein isothiocyanate, and Oregon Green)-conjugated streptavidin, but few fluorescent signals were obtained in the 100-nm thin sections of Epon-embedded cells (data not shown). This was probably because of the green autofluorescence of epoxy resins. We examined tetramethylrhodamine and rhodamine red instead of DyLight 549 for this in-resin CLEM using miniTurbo. However, the results were poor probably because of the chemical properties of these fluorophores or the stability of the conjugation. For in-resin CLEM of Epon-embedded cells, fluorophores should be resistant to osmium tetroxide (1–2%), 100% ethanol, and polymerization at 60 °C for 72 h. Fluorophores for in-resin CLEM of Epon-embedded cells should be screened and developed based on these criteria in the future.

We showed that this in-resin CLEM method with miniTurbo was more stable and gave brighter fluorescent signals than that with mCherry2 (Fig. 4). However, in contrast to in-resin CLEM using ‘fluorescent proteins’, this in-resin CLEM requires cell-permeabilization. Therefore, this in-resin CLEM with miniTurbo must determine the conditions for permeabilization without significant loss of ultrastructures after fixation of the cells. We overcame this limitation by treatment with 50 µg/ml digitonin (or 50 µg/ml digitonin and 0.02% Trion X-100 for the nucleus) after fixation and achieved this in-resin CLEM without significant loss of ultrastructures (Figs. 3, 5, and 6).

In the case of in-resin CLEM of Epon-embedded cells expressing miniTurbo, a reduction of the fluorescent signal was observed in the areas of the mitochondria (Fig. 3B). For these conditions, we employed digitonin for cell-permeabilization. Digitonin is a mild detergent that permeabilizes plasma membranes mainly, and it is possible that less digitonin was able to permeabilize the outer membranes of mitochondria and nucleus. Therefore, a reduction of fluorescent signal inside mitochondria was observed.

We showed that fluorescent signals based on biotinylated proteins by mtActA-mTurbo were well correlated with mitochondrial structures in the electron microscopic images (Fig. 5 and Supplementary Fig. 3). However, we have to take care that the fluorescent signals in this method are derived from biotinylated proteins (including self-biotinylated miniTurbo) modified by miniTurbo, not from self-biotinylated miniTurbo alone. Biotin ligases used for the proximity labeling including miniTurbo are known to biotinylate proximal proteins (labeling radius < 10 nm)^25^. It is possible that the subcellular localization of some biotinylated proteins changes as time passes, resulting in diffusive fluorescent signals from the biotinylated proteins. Therefore, it is important to optimize the concentration of biotin and the time for biotinylation when miniTurbo fused with a protein of interest is used for this method.

We are interested in application of affinity-labeling for two (or multi) color in-resin CLEM of Epon-embedded samples. In this study, we developed proximity labeling-based in-resin CLEM using miniTurbo. But that is not enough for affinity-based ‘multicolor’ in-resin CLEM, because fluorescent signals are dependent only on the biotin-streptavidin reaction. For multicolor labeling, we are trying other affinity-based reactions suitable for in-resin CLEM of Epon-embedded cells. Future studies will be required to solve these problems for affinity-labeling based multicolor in-resin CLEM of Epon-embedded cells.

## Methods

### Cells, media, and materials

HeLa cells were obtained from the American Type Culture Collection and were cultured in Dulbecco’s Modified Eagle’s Medium (DMEM, Nacalai Tesque, #08458-45) containing 10% fetal bovine serum (Equitech Bio, #268-1). For biotinylation of intracellular proteins by biotin ligase, (+)-biotin (Fujifilm Wako Chemicals, #029-08713) was added to the medium at the indicated concentration (if not indicated, 300 µM) and incubated at 37 °C for 40 min. FuGENE HD Transfection Reagent was used to introduce the plasmid into cells (Promega, E2311). For high fidelity polymerase chain reaction, a KOD One PCR Master Mix (Toyobo, KMM-201) was used.

To generate an expression plasmid for miniTurbo^15^ under the control of the CAG promoter, a DNA fragment (Supplementary Data) was synthesized generated by Integrated DNA Technologies (Iowa), and the open reading frame of mCherry2 of pCAG-mCherry2-G (Addgene #164162)^7^ was replaced with a DNA fragment of miniTurbo (pCAG-mTurbo-G, Addgene #187176). For the expression of mitochondria-targeting miniTurbo under the control of the CAG promoter, a DNA fragment encoding miniTurbo fused with a mitochondria-targeting signal^20^ of the *ActA* gene of *Listeria monocytogenes* was introduced into pCAG (pCAG-mTurbo-mito, Addgene #187177). For the expression of histone H2B fused with miniTurbo under the control of the CAG promoter, a DNA fragment encoding histone H2B was generated by a KOD one PCR Master Mix using primer sets (H2B-cagarm-Nhe-ATG-F: GTG CTG TCT CAT CAT TTT GGC AAA GCT AGC GCC ACC ATG CCA GAG CCA GCG AAG TCT GCT CCC GCC, H2B-mTurbo-atgarm-Rv: CCA CCA GTC ATA GAG GCC ATC TTA GCG CTG GTG TAC TTG GTG ATG GCC TT) and genomic DNA isolated from HEK293 cells as a template. A DNA fragment encoding histone H2B was introduced into a NheI-site of pCAG-mTurbo-G by a NEBuilder HiFi DNA Assembly Master Mix (New England Biolabs, E2621) (pCAG-H2B-mTurbo, Addgene #187178).

### DNA transfection and biotin treatment

HeLa cells were seeded on a 3.5 mm culture dish or cover slips in 12-well plates, incubated for 8 h, and transfected with miniTurbo or miniTurbo-ActA plasmid DNA using FuGENE HD (Promega, E2311) according to the manufacturer’s instructions. After incubation for 16–18 h, the cells were incubated in the (+)-biotin containing medium at the indicated time points. After biotin treatment, the cells were incubated in fresh medium to remove intracellular free biotin for 40 min before the following experiments.

### Sample-preparation, fluorescence microscopy, and electron microscopy for in-resin CLEM

Cells expressing miniTurbo and its fusion proteins were prefixed with a fixation solution containing 4% paraformaldehyde and 0.25% glutaraldehyde at 4 °C for 30 min. The fixed cells were washed three times with HB solution (Fujifilm Wako Chemicals, # 080-10591)^7,9,26^ and permeabilized with HB containing 50 µg/ml digitonin for 10 min for miniTurbo and mtActA-mTurbo (Figs. 1, 2, 3, 4, and 5) and additionally 0.02% Triton-X100 for 5 min for histone H2B fused with miniTurbo (Fig. 6). The cells were incubated in HB solution containing 1% bovine serum albumin. After washing the cells with HB solution three times, the cells were incubated in HB solution containing 1 µg/ml of DyLight 549-conjugated streptavidin (VECTOR Lab, # SA-5549-1) at room temperature for 30 min. Cells were post-fixed in 2% osmium tetroxide at 4 °C for 30 min (for miniTurbo and mtActA- mTurbo) or 1% osmium tetroxide at 4 °C for 10 min (miniTurbo-H2B) and were washed three times with HB solution, incubated in TUK Solution for multicolor (Fujifilm Wako Chemicals, # 208-21161)^7,9,26^ at 4 °C for 10 min to recover the fluorescent signal decreased by osmium tetroxide treatment, fixed with fixation solution containing 2.5% glutaraldehyde at 4 °C for 3 h, and washed three times with HB solution. Cells were washed with HB solution after treatment with TUK Solution to produce clearer electron microscopic images. Cells were dehydrated with a graded series of ethanol and embedded in Epon812 (Oken Shoji) at 60 °C for 72 h. Ultrathin sections (100 nm) were cut with an Ultramicrotome UC6 (Leica) and placed on 12-mm circular glass cover slips. The cover slips were pre-coated with Pt/Au (about 2.5-nm thickness) using an Ion Sputter E-1010 (Hitachi) to prevent the undesirable accumulation of negative charge from the incident electron beam on the surface of non-conductive specimens during scanning electron microscopy. Sections were observed in TUK Solution for multicolor using a BZ-X810 fluorescence microscope (Keyence). Fluorescence of fluorophore in the cells of Epon-embedded samples was observed in HB solution. After observation, the thin sections were washed with distilled water, dried overnight at room temperature, stained with uranyl acetate and lead citrate, and observed via a Helios NanoLab 660 scanning electron microscope (FEI). The electron microscopic images were obtained using a backscattered electron detector (circular backscatter detector) at a voltage of 2.0 kV and a current of 0.4 nA as described previously^9^.

### Data analyses and statistics

Fluorescent intensities in the fluorescent images were evaluated using ImageJ/Fiji software. Differences between two groups were assessed using two-tailed unpaired Student t test with Welch's correction. Comparisons for more than two groups were assessed using one-way ANOVA with the Tukey–Kramer test.

## Supplementary Information

Supplementary Information.

## Data Availability

The DNA sequences encoding the ORFs used in this study were listed in Supplemental data. The plasmids used in this study were deposited with Addgene (https://www.addgene.org/plasmids/articles/28228846/) (pCAG-mTurbo-G, Addgene #187176; pCAG-mTurbo-mito, Addgene #187177; pCAG-H2B-mTurbo, Addgene #187178).
