# Supplementary material for: In-resin CLEM of Epon-embedded cells using proximity labeling
Source: Sci Rep. 2022 Jul 1;12:11130. doi: 10.1038/s41598-022-15438-6 (PMC9249884; doi:10.1038/s41598-022-15438-6)
Supplement: Supplementary file 1 — Supplementary Information. [file 41598_2022_15438_MOESM1_ESM.pdf]

In-resin CLEM of Epon-embedded cells using proximity labeling

Takahito Sanada[1], Junji Yamaguchi[2], Yoko Furuta[1], Soichiro Kakuta[2], Isei Tanida[1]*, and Yasuo Uchiyama[1]*

[1]Department of Cellular and Molecular Neuropathology, Juntendo University Graduate School of Medicine, Tokyo, Japan

[2]Laboratory of Morphology and Image Analysis, Biomedical Research Core Facilities, Juntendo University Graduate School of Medicine, Tokyo, Japan

*Corresponding author:

Isei Tanida and Yasuo Uchiyama

Email: tanida@juntendo.ac.jp (I.T.) and y-uchi@juntendo.ac.jp (Y.U.)

Tel: +81-3-3813-3111 ex 3601 (I.T.)

Supplementary Fig. 1. Intracellular proteins in the cultured cells were biotinylated by miniTurbo in a time-dependent manner. (**A**) HeLa cells expressing miniTurbo and mtActA-mTurbo were incubated for the indicated times (10, 20, 40, 80, 160 min) in culture medium containing 300 μM biotin. After preparation of the cell lysate, total proteins were separated using sodium dodecyl sulfate polyacrylamide gel electrophoresis and transferred to a polyvinylidene difluoride membrane. Biotinylated proteins and miniTurbo (or mtActA-mTurbo), respectively, in the lysate were detected by Western blotting with rabbit polyclonal anti-biotin (Jackson ImmunoResearch) and mouse monoclonal anti-BirA (Novus Biologicals) antibodies as the first antibodies. As second antibodies, the respective anti-rabbit and anti-mouse antibodies conjugated with horseradish peroxidase (Jackson Immuno Research) were employed. Chemiluminescent signals in the membrane were detected with a chemiluminescence imaging system, Fusion FX (Vilber Bio Imaging), using a SuperSignal West Dura Extended Duration Substrate (ThermoFisher). As a loading control, ß-actin was detected using anti-ß-actin antibody. (**B**) Chemiluminescent signals in the blots of miniTurbo and mtActA-mTurbo of **A** were analyzed and quantified using BIO-1D software (Vilber Bio Imaging) on a Fusion FX imaging system. Relative intensity (AU) is the ratio of the signal with anti-biotin antibody to the signal with anti ß-actin antibody (mean ± SEM, n = 3 per group). Left graph, miniTurbo; Right graph, mtActA-mTurbo. **P* value < 0.05, ***P* value < 0.01. (**C**) The analyzed values in **B** quantified using BIO-1D software are shown as a Table. (**D**) The raw uncropped images used in **A** are shown.

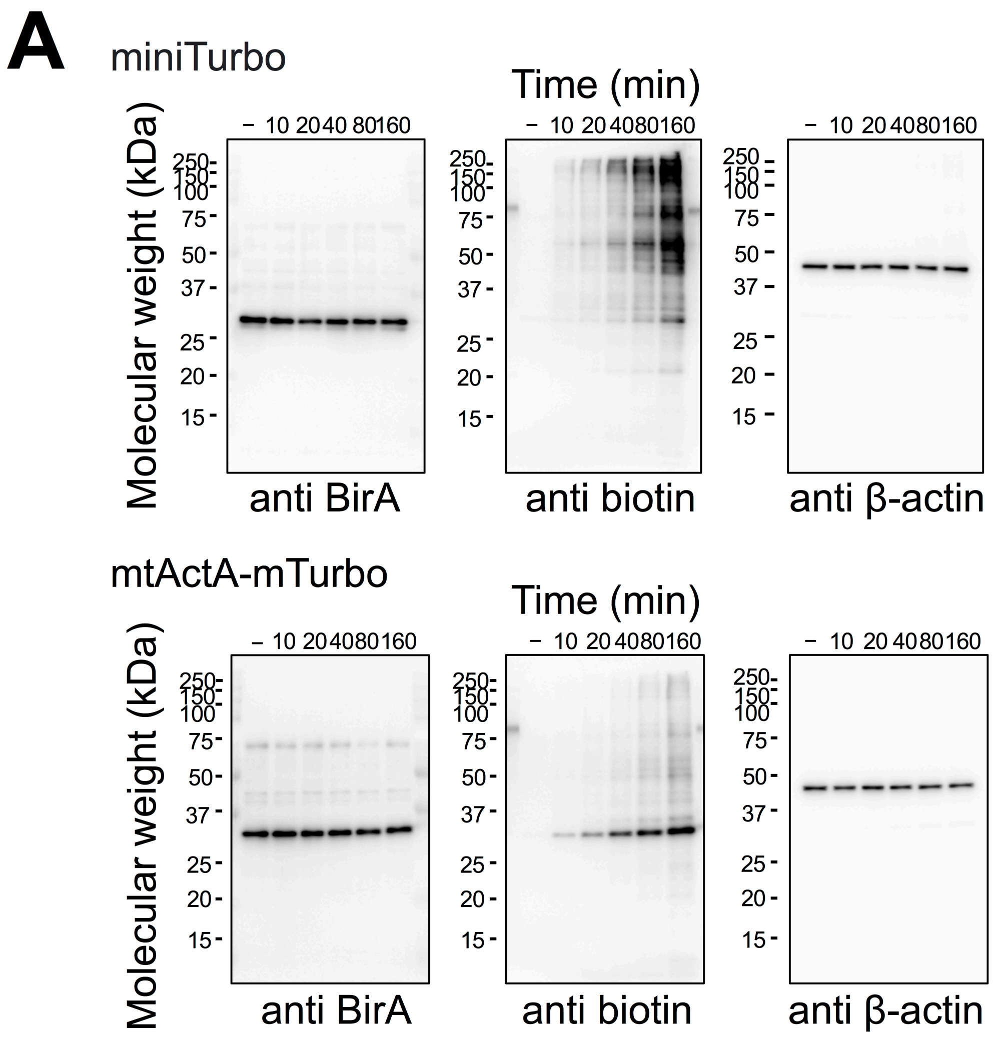
A
miniTurbo
Time (min)
– 10 20 40 80 160
Molecular weight (kDa)
250
150
100
75
50
37
25
20
15
anti BirA
anti biotin
anti β-actin
mtActA-mTurbo
Time (min)
– 10 20 40 80 160
Molecular weight (kDa)
anti BirA
anti biotin
anti β-actin

B

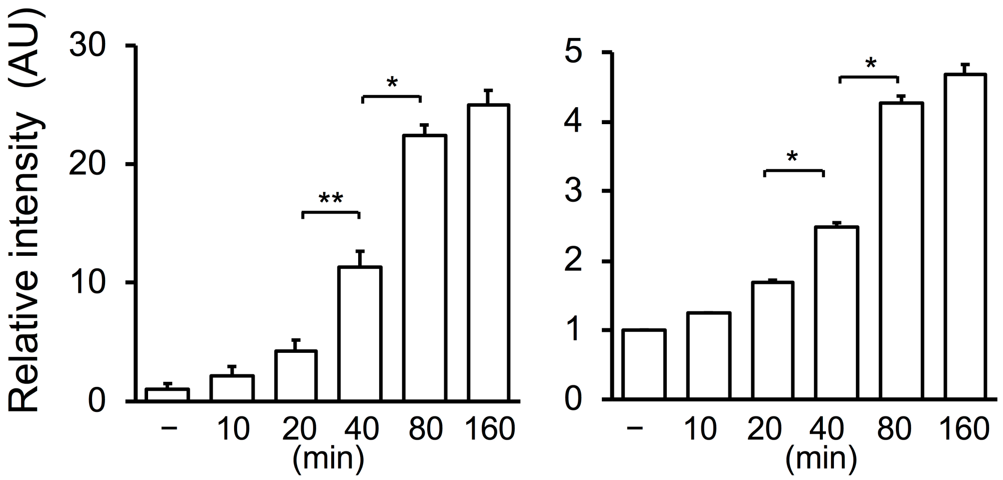

C

	The average value of the volume ± SEM	
	miniTurbo	β-actin
— (min)	7.80E+06 ± 6.48E+06	2.81E+07 ± 2.82E+06
10	1.57E+07 ± 8.76E+06	2.56E+07 ± 1.87E+06
20	3.47E+07 ± 1.15E+07	2.92E+07 ± 1.84E+06
40	8.98E+07 ± 1.84E+07	2.87E+07 ± 5.89E+05
80	2.13E+08 ± 2.56E+07	3.40E+07 ± 3.46E+06
160	2.85E+08 ± 3.82E+07	4.09E+07 ± 3.74E+06

	The average value of the volume ± SEM	
	mt-mTurbo	β-actin
— (min)	4.45E+07 ± 1.34E+07	1.65E+07 ± 1.88E+06
10	5.86E+07 ± 1.20E+07	1.76E+07 ± 1.49E+06
20	7.37E+07 ± 1.07E+07	1.62E+07 ± 2.22E+06
40	1.09E+08 ± 1.08E+07	1.62E+07 ± 2.51E+06
80	1.77E+08 ± 1.44E+07	1.54E+07 ± 2.14E+06
160	2.26E+08 ± 1.80E+07	1.80E+07 ± 1.74E+06

D

For supplementary Fig. 1A

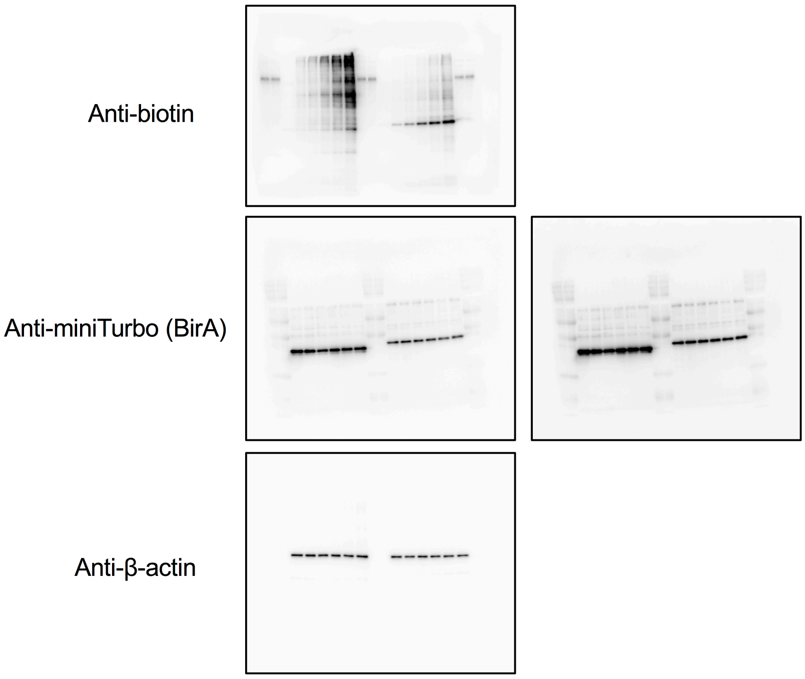

Supplementary Fig.2. Biotinylated proteins in HeLa cells expressing mtActA-mTurbo fusion protein were localized to mitochondria. HeLa cells expressing mtActA-mTurbo were cultured, and 300 µM biotin was added to the medium. After incubation at 37ºC for 40 min, cells were fixed with 4% paraformaldehyde. Intracellular biotinylated proteins were labeled using DyLight549-conjugated streptavidin (**mt-mTurbo**). **Tom20**, a mitochondrial marker, was stained by anti-Tom20 antibody. Nuclei were stained with 4’,6-diamidino-2-phenylindole (**DAPI**). Fluorescence was detected with a LSM880 confocal fluorescent microscopy (Zeiss). The “**merge**” images are merged images of a fluorescence image of **mt-mTurbo** (green pseudo color), **Tom20** (red pseudo color), and **DAPI** (blue pseudo color). The images in the lower panels indicate 5x magnification of images corresponding to the boxed area in the **merge** image in the upper panels. Colocalization of Tom20-positive signals with fluorescent signals derived from biotinylated proteins mediated by mt-mTurbo was evaluated as a Pearson correlation coefficient value using ImageJ/Fiji software with the Coloc2 plugin (https://imagej.net/plugins/coloc-2). The mean ± SEM of the Pearson correlation coefficient value was 0.82 ± 0.012 (n = 12), indicating strong colocalization between the two signals.

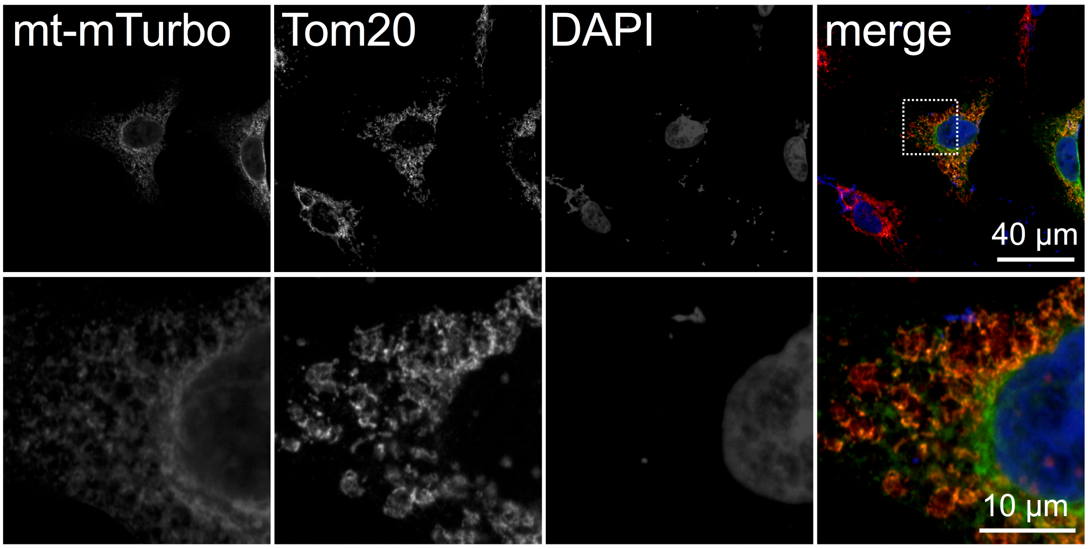

Supplementary Fig. 3. Fluorescent signals in cells expressing mtActA-mTurbo were well-correlated with the ultrastructures of mitochondrial outer membrane in the electron microscopic images. HeLa cells expressing mtActA-mTurbo were incubated in the presence of 300 μM biotin for 40 min, labeled with DyLight549-streptavidin, post-fixed with osmium tetroxide, embedded in epoxy resin, and sliced to sections of 100 nm. Fluorescent images were obtained with a BZ-X810 fluorescence microscope. The electron microscopic images were obtained via a Helios NanoLab 660 scanning electron microscope at 8000x magnification. An in-resin CLEM analysis was performed for two different fields per cell (n = 3), and the ratio of fluorescence-positive mitochondrial membrane was quantified via a point counting method. A total of 50 × 71 grid points were drawn on the in-resin CLEM images, and the presence or absence of a fluorescence signal at each grid point that overlapped with the mitochondrial outer membrane was counted and converted to a percentage (%).

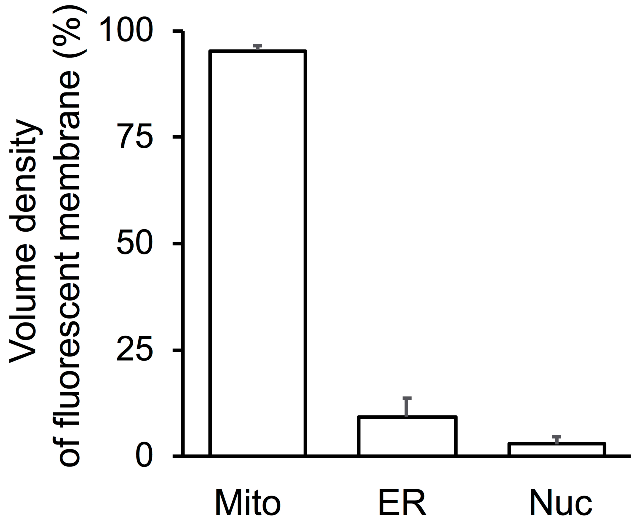

Supplementary Fig. 4. Schematic diagram of the in-resin CLEM method with proximity labeling.

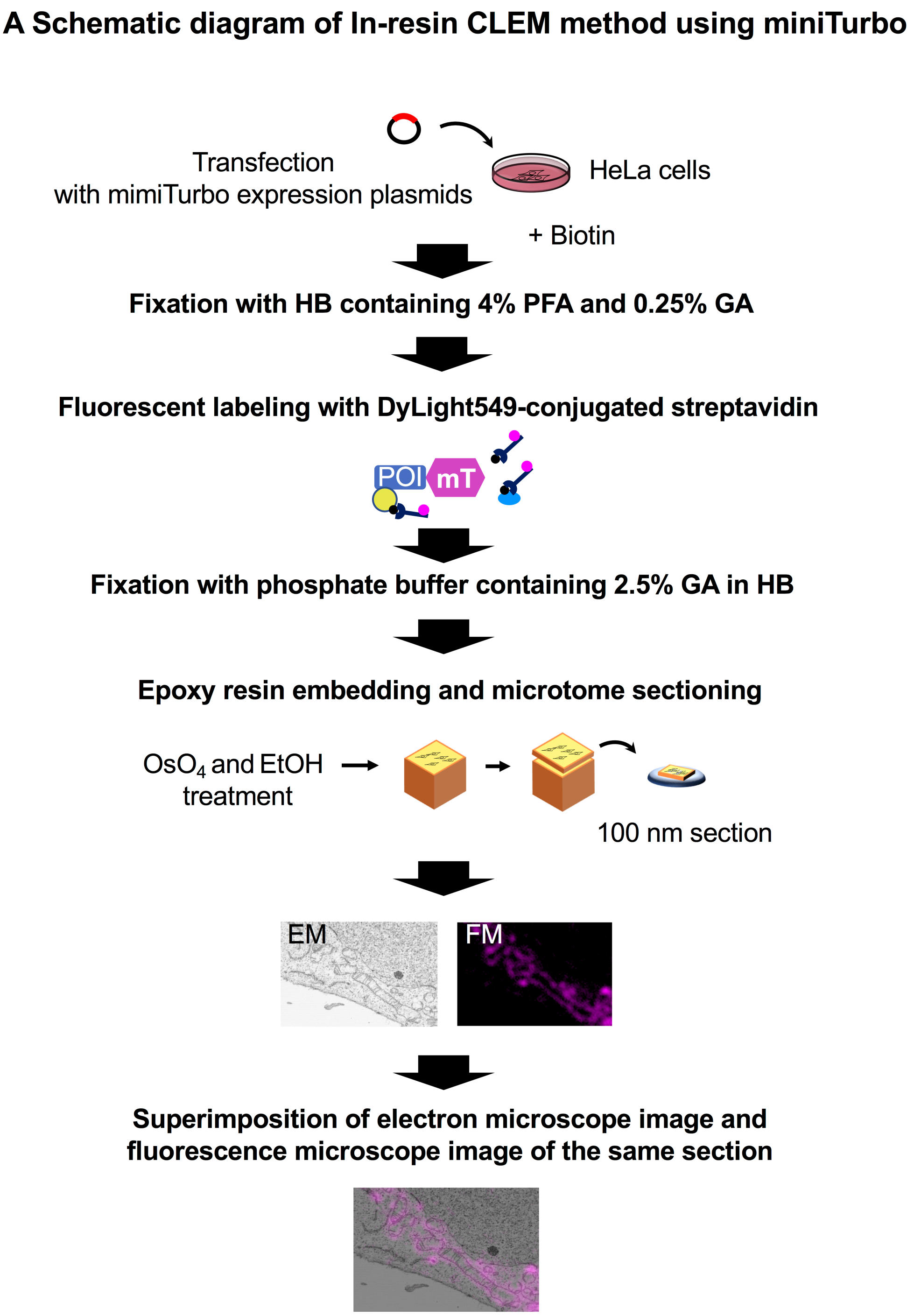

Supplementary Data. The DNA sequences encoding the ORFs used in this study.

miniTrubo	ATGGCCTCTATGACTGGTGGTCAACAAATGGGCCGGGGAAGCGAATTTATGATT CCTCTTCTGAACGCAAAGCAAATACTGGGTCAGCTCGACGGGGGATCAGTCGCT GTTTTGCCTGTTGTTGACTCTACTAATCAATATCTCTTGGATCGAATCGGTGAA CTCAAATCTGGCGATGCCTGTATCGCCGAATACCAACAGGCAGGTAGAGGTTCT AGAGGGAGAAAATGGTTCTCCCCGTTTGGTGCCAATCTCTATTTGAGTATGTTT TGGCGGCTTAAGCGAGGACCGGCGGCTATCGGTCTCGGACCTGTTATTGGCATC GTCATGGCCGAGGCGCTTAGGAAACTTGGAGCCGATAAAGTCAGGGTCAAGTGG CCAAATGATTTGTATTTGCAAGACAGAAAGCTCGCAGGCATCTTGGTTGAGCTT GCTGGGATAACGGGCGACGCAGCACAAATAGTCATTGGTGCCGGAATCAATGTC GCTATGCGCCGGGTTGAAGAAAGTGTCGTGAACCAAGGTTGGATAACTCTTCAA GAAGCGGGTATTAACCTCGATCGCAATACTCTCGCCGCTATGCTCATCAGGGAA CTGCGAGCGGCCCTGGAGTTGTTCGAGCAAGAAGGTCTCGCTCCCTATTTGTCA CGCTGGGAAAAGTTGGATAATTTCATTAACCGACCAGTCAAACTTATTATTGGC GATAAAGAAATATTTGGGATCTCACGCGGTATCGATAAGCAAGGCGCATTGTTG CTTGAACAGGATGGAGTGATTAAACCATGGATGGGAGGTGAGATCAGCCTTAGA AGTGCGGAAAAGAAA
H2B-miniTurbo	ATGCCAGAGCCAGCGAAGTCTGCTCCCGCCCCGAAAAAGGGCTCCAAGAAGGCG GTGACTAAGGCGCAGAAGAAAGGCGGCAAGAAGCGCAAGCGCAGCCGCAAGGAG AGCTATTCCATCTATGTGTACAAGGTTCTGAAGCAGGTCCACCCTGACACCGGC ATTTCGTCCAAGGCCATGGGCATCATGAATTCGTTTGTGAACGACATTTTCGAG CGCATCGCAGGTGAGGCTTCCCGCCTGGCGCATTACAACAAGCGCTCGACCATC ACCTCCAGGGAGATCCAGACGGCCGTGCGCCTGCTGCTGCCTGGGGAGTTGGCC AAGCACGCCGTGTCCGAGGGTACTAAGGCCATCACCAAGTACACCAGCGCTAAG ATGGCCTCTATGACTGGTGGTCAACAAATGGGCCGGGGAAGCGAATTTATGATT CCTCTTCTGAACGCAAAGCAAATACTGGGTCAGCTCGACGGGGGATCAGTCGCT GTTTTGCCTGTTGTTGACTCTACTAATCAATATCTCTTGGATCGAATCGGTGAA CTCAAATCTGGCGATGCCTGTATCGCCGAATACCAACAGGCAGGTAGAGGTTCT AGAGGGAGAAAATGGTTCTCCCCGTTTGGTGCCAATCTCTATTTGAGTATGTTT TGGCGGCTTAAGCGAGGACCGGCGGCTATCGGTCTCGGACCTGTTATTGGCATC GTCATGGCCGAGGCGCTTAGGAAACTTGGAGCCGATAAAGTCAGGGTCAAGTGG CCAAATGATTTGTATTTGCAAGACAGAAAGCTCGCAGGCATCTTGGTTGAGCTT GCTGGGATAACGGGCGACGCAGCACAAATAGTCATTGGTGCCGGAATCAATGTC GCTATGCGCCGGGTTGAAGAAAGTGTCGTGAACCAAGGTTGGATAACTCTTCAA GAAGCGGGTATTAACCTCGATCGCAATACTCTCGCCGCTATGCTCATCAGGGAA CTGCGAGCGGCCCTGGAGTTGTTCGAGCAAGAAGGTCTCGCTCCCTATTTGTCA

	CGCTGGGAAAAGTTGGATAATTTCATTAACCGACCAGTCAAACTTATTATTGGC GATAAAGAAATATTTGGGATCTCACGCGGTATCGATAAGCAAGGCGCATTGTTG CTTGAACAGGATGGAGTGATTAAACCATGGATGGGAGGTGAGATCAGCCTTAGA AGTGCGGAAAAGAAAT
miniTurbo-mtActA	ATGGCCTCTATGACTGGTGGTCAACAAATGGGCCGGGGAAGCGAATTTATGATT CCTCTTCTGAACGCAAAGCAAATACTGGGTCAGCTCGACGGGGGATCAGTCGCT GTTTTGCCTGTTGTTGACTCTACTAATCAATATCTCTTGGATCGAATCGGTGAA CTCAAATCTGGCGATGCCTGTATCGCCGAATACCAACAGGCAGGTAGAGGTTCT AGAGGGAGAAAATGGTTCTCCCCGTTTGGTGCCAATCTCTATTTGAGTATGTTT TGGCGGCTTAAGCGAGGACCGGCGGCTATCGGTCTCGGACCTGTTATTGGCATC GTCATGGCCGAGGCGCTTAGGAAACTTGGAGCCGATAAAGTCAGGGTCAAGTGG CCAAATGATTTGTATTTGCAAGACAGAAAGCTCGCAGGCATCTTGGTTGAGCTT GCTGGGATAACGGGCGACGCAGCACAAATAGTCATTGGTGCCGGAATCAATGTC GCTATGCGCCGGGTTGAAGAAAGTGTCGTGAACCAAGGTTGGATAACTCTTCAA GAAGCGGGTATTAACCTCGATCGCAATACTCTCGCCGCTATGCTCATCAGGGAA CTGCGAGCGGCCCTGGAGTTGTTCGAGCAAGAAGGTCTCGCTCCCTATTTGTCA CGCTGGGAAAAGTTGGATAATTTCATTAACCGACCAGTCAAACTTATTATTGGC GATAAAGAAATATTTGGGATCTCACGCGGTATCGATAAGCAAGGCGCATTGTTG CTTGAACAGGATGGAGTGATTAAACCATGGATGGGAGGTGAGATCAGCCTTAGA AGTGCGGAAAAGAAATCCGGACTCACCGGTGGAGGCGGTTCAGGCGGAGGTGGC TCTGGCGGTGGCGGATCGAGATCTCGAGCTCAAGCTTCGAATTCTAAACTAATT GCTAAAAGTGCAGAAGACGAAAAAGCGAAGGAAGAACCAGGGAACCATACGATC GTAATTCTTGCAATGTTAGCTATTGGCGTGTTCTCTTTAGGGGCGTTTATCAAA ATTATTCAATTAAGAAAAAATAATTAA

:
